# Supplementary material for: Rising food prices and poverty in Pakistan
Source: PLoS One. 2023 Nov 16;18(11):e0292071. doi: 10.1371/journal.pone.0292071 (PMC10653518; doi:10.1371/journal.pone.0292071)
Supplement: Attachment — Submitted filename: Reviewer Response 1.docx [file pone.0292071.s002.docx]

**Journal Requirements:**

1) **Please ensure that your manuscript meets PLOS ONE's style requirements, including those for file naming.**

**Response:** Thank you dear editor for pointing out these flaws in the formatting of manuscript. We have done all the style requirements in the revised manuscript.

2) **In your Data Availability statement, you have not specified where the minimal data set underlying the results described in your manuscript can be found. PLOS defines a study's minimal data set as the underlying data used to reach the conclusions drawn in the manuscript and any additional data required to replicate the reported study findings in their entirety. All PLOS journals require that the minimal data set be made fully available.**

**Response:** We have provided the data in supporting file so that researcher can replicate the results.

**Please ensure that you include a title page within your main document. You should list all authors and all affiliations as per our author instructions and clearly indicate the corresponding author.**

**Response:** Yes, we have included the title page with all the necessary requirements.

3) **We note you have included a table to which you do not refer in the text of your manuscript. Please ensure that you refer to Table 1 in your text; if accepted, production will need this reference to link the reader to the Table.**

**Response:** We have provided the reference of Table 1 in the body of the text.

4) **Please review your reference list to ensure that it is complete and correct. If you have cited papers that have been retracted, please include the rationale for doing so in the manuscript text, or remove these references and replace them with relevant current references. Any changes to the reference list should be mentioned in the rebuttal letter that accompanies your revised manuscript. If you need to cite a retracted article, indicate the article’s retracted status in the References list and also include a citation and full reference for the retraction notice.**

**Response:** We have complete the reference list in the revised manuscript.

**Reviewer # 1 (Review of the manuscript PONE-D-23-03624)**

Rising food prices and their impacts on poverty is a very pertinent issue for Pakistan these days. The paper attempts to explore this relationship in an articulated way. However, I have few minor suggestions.

1) **The abstract of the paper needs to be improved.**

**Response:** We are thankful to the reviewer for useful comments and highlighting this aspect. We have improved the abstract of the paper to our best.

2) **Descriptive analysis should be explained in the results section rather than Methodology section.**

**Response:** As per reviewer comment, we explained the descriptive analysis in the result section. Please see the line 198 to onward.

3) **In Tables 3 & 4, the variables need to be renamed for better understanding of the table.**

**Response:** As per reviewer comment, we have renamed the variables.

**Reviewer # 2 (Review of the manuscript PONE-D-23-03624)**

1) **Revise the abstract and make it more crisp and interesting.**

**RESPONSE:** As per reviewer’s suggestion, we have revised the abstract.

2) **A stronger motivation and economic intuition for the topic should be provided in introduction section. Scarcity does not mean necessity. You need to prove your paper is valuable enough and distinguish from others.**

**RESPONSE:** We are really thankful to the reviewer for the valuable comment. We have incorporated the suggestions in introduction section.

3) **Please compare and contrast the relevant studies from existing literature in the results and discussion section.**

**RESPONSE:** As per reviewer comment, we have included the relevant studies for comparison in the discussion section.

4) **Based upon your empirical results, propose concrete policy recommendations and provide future directions for researchers.**

**RESPONSE**: We have provided appropriate policy recommendations and future direction to the researchers. Please see the line 443 and section Policy implications and recommendation
